# The interpretation of very high frequency band of instantaneous pulse rate variability during paced respiration

**DOI:** 10.1186/1475-925X-13-46

**Published:** 2014-04-21

**Authors:** Chia-Chi Chang, Hung-Yi Hsu, Tzu-Chien Hsiao

**Affiliations:** 1 Biomedical Electronics Translational Research Center and Biomimetic Systems Research Center, National Chiao Tung University, Taiwan, ROC; 2 Department of Neurology, Chung Shan Medical University, Taiwan, ROC; 3 Section of Neurology, Department of Internal Medicine, Tungs’ Taichung Metro Harbor Hospital, Taiwan, ROC; 4 Institute of Biomedical Engineering, National Chiao Tung University, Taiwan, ROC; 5 Department of Computer Science, National Chiao Tung University, 1001 University Road, Hsinchu 300 Taiwan, ROC

**Keywords:** Pulse rate variability, Hilbert-Huang transform, Empirical mode decomposition

## Abstract

**Background:**

Pulse rate (PR) indicates heart beat rhythm and contains various intrinsic characteristics of peripheral regulation. Pulse rate variability (PRV) is a reliable method to assess autonomic nervous system function quantitatively as an effective alternative to heart rate variability. However, the frequency range of PRV is limited by the temporal resolution of PR based on heart rate and it is further restricted the exploration of optimal autoregulation frequency based on spectral analysis.

**Methods:**

Recently, a new novel method, called instantaneous PRV (iPRV), was proposed. iPRV breaks the limitation of temporal resolution and extends the frequency band. Moreover, iPRV provides a new frequency band, called very high frequency band (VHF; 0.4-0.9 Hz).

**Results:**

The results showed that the VHF indicated the influences of respiratory maneuvers (paced respiration at 6-cycle and 30-cycle) and the nonstationary condition (head-up tilt; HUT).

**Conclusions:**

VHF is as a potential indication of autoregulation in higher frequency range and with peripheral regulation. It helps for the frequency exploration of cardiovascular autoregulation.

## Background

Heart beat rhythm is regulated by autonomic nervous system (ANS), and its variability, called heart rate variability (HRV), reflects ANS activities and provides meaningful information for clinical intervention in cardiovascular disease [1]. The pulse rhythm of blood pressure waveform, driven by left ventricular contractions, indicates heart beat rhythm and its fluctuations are modulated by autonomic function [2]. It had been examined that pulse rate variability (PRV) is a surrogate measurement of HRV during nonstationary conditions, such as head-up tilt (HUT) [3], and presents the status of cardiovascular autoregulation [4]. But the transmission of blood pressure pulse depends on the elasticity of the vessels. Moreover, PRV is influenced by volumetric and oxygenation changes in the peripheral microvasculature and by the peripheral tissue responsiveness, and contains much more complex information than HRV.

In order to quantitatively assess autoregulation function through PRV, several spectral analysis methods were applied, such as fast Fourier transform (FFT) and discrete wavelet transform (DWT). Conventional spectral analysis of PRV (and HRV) contains three major frequency bands in quantitative ANS assessment, including very low frequency band (VLF; 0.01-0.04 Hz), low frequency band (LF; 0.04-0.15 Hz), and high frequency band (HF; 0.15-0.4 Hz), in short-term recording (5–10 minutes) [4]. Each frequency band indicates specific ANS activities, such as HF related to parasympathetic nervous activities (PNS) and LF related to the modulation of sympathetic nervous activities (SNS) and PNS. The frequency range of PRV spectrum depends on temporal resolution of the pulse rate series in time domain, and it limits the indication of intrinsic nonstationary fluctuations in high frequency range based on PRV analysis [5]. Though several literatures investigated that the nonlinear methods, such as multiscale entropy or higher order spectral analysis, provide nonlinear estimator for ANS activities evaluation, beat-to-beat intervals as target signal still limit the indication of these methods and cannot present continuous-time nonstationary intrinsic characteristics of pulse rhythm, such as its intrawave frequency modulation, in much higher frequency band (>0.5 Hz). In order to breakthrough this limitation and explore those intrinsic fluctuations in much higher frequency band caused by autoregulation, the nonstationary feature extraction method should be performed to sift the continuous-time intrinsic characteristics.

Recently, a new novel method, called empirical mode decomposition (EMD), was proposed and it has good capability for continuous-time nonstationary intrinsic fluctuations extraction [6]. It had been examined that the reliable nonstationary intrinsic characteristics were extracted from blood pressure waveform by EMD [7], but the usefulness of these characteristics still needs further examination. The study proposed a method, called instantaneous PRV (iPRV) [8], and investigated that the nonstationary intrinsic characteristics of blood pressure waveform extracted by EMD help for PRV spectral analysis with extended frequency range. iPRV extracts the intrinsic fluctuation, which indicates pulse rhythm, from blood pressure waveform based on EMD and estimates the pulse rhythm series, called instantaneous pulse rate (iPR) series, by instantaneous frequency projecting based on normalized Hilbert transform (NHT). By applying EMD and NHT, so-called Hilbert-Huang transform (HHT), the intrinsic characteristics of pulse rhythm had been explored and it improves the temporal resolution of target signal of spectral analysis method, which is equal to the sampling rate of the measurement, and extends the frequency range of the spectrum. It had been examined that PRV can be evaluated by iPRV [8]. Moreover, iPRV breaks through the limitation of the frequency range of PRV and provides a new frequency band, named very high frequency band (VHF; 0.4-0.9 Hz) [8]. Though several studies investigated that the spectral power of VHF of HRV is a reliable evaluation index of cardiovascular diseases, such as chronic heart failure [9] and coronary artery disease [10], the clinical interpretation of the spectral power of VHF of iPRV is still unknown. iPRV contains various nonstationary intrinsic fluctuations, including the influence of myogenic vascular function, vessel compliance, and peripheral tissue responses, which are not in HRV. The potential clinical indication of VHF of iPRV needs further examination.

It had been examined that the specific ANS activities were induced during active paced respiration [11-13] and the difference between supine baseline and paced respiration has potential to indicate the autoregulation function. The aim of this study is to 1) examine the potential indication of VHF of iPRV by paced respirations, and 2) interpret the physiological meaning of VHF of iPRV during different nonstationary conditions.

## Methods

### Subjects and data collection

All measurements were performed in a quiet temperature controlled room. The beat-to-beat arterial blood pressure (ABP) was recorded by Task Force® Monitor equipped with a servo-controlled plethysmography (CNSystems, Medizintechnik AG, Graz, Austria) with a sampling frequency of 200 Hz. The end-tidal CO_2_ (EtCO_2_; RespSenseTM EtCO_2_, Nonin Medical Inc.) concentration were recorded simultaneously as the external signal of Task Force® Monitor. All raw data were exported from Task Force® Monitor and the offline analysis was performed after the experiments.

Fifteen healthy subjects (male: 9; age: 43 ± 17), who did not have the history of cardiovascular disease, participated this study. All recruited subjects were asked to rest quietly in supine position for 10 minutes. While all recorded signals were stabilized, a 10-minute baseline recording was performed under spontaneous breathing. Then, all recruited participants were asked to breathe following the examiner’s verbal instruction at a rate of 6 cycles per minute (6-cycle breathing) and at 30 cycles per minute (30-cycle breathing). Each test was performed once for 2 minutes. There was a 5-minute rest between each test. At the end, the participants were tilting up passively on the automatic tilting table test and kept in HUT for 10 minutes. This study was approved by institutional review board of Tungs’ Taichung Metro Harbor Hospital. Informed consent was obtained from all participants before the experiment.

### Empirical mode decomposition

The nonstationary intrinsic feature extraction of HHT is called EMD. During EMD, The intrinsic mono-components, called intrinsic mode functions (IMFs), are extracted by sifting process, as an iteratively detrending operation. The essence of sifting process is based on energy associated extraction at each time scale. The time scale is determined by the locations of local extrema. The sifting process contains several steps. First, the local extrema of the time series *x(t)* are identified by peak-valley detection. The upper envelope *U(t)* and lower envelope *L(t)* are generated by cubic spline interpolation according to the local maxima and the local minima, respectively. Both *U(t)* and *L(t)* cover *x(t)* in current time scale. The trend in current time scale is computed by calculating the mean of *U(t)* and *L(t)*.

(1) $$m\left(t\right)=\left(U\left(t\right)+L\left(t\right)\right)/2$$

The sifting process subtracted the trend from original time series *x(t)*, as detrending operation.

(2) $$h_{1}\left(t\right)=x\left(t\right)-m_{1}\left(t\right)$$

And the subtraction was iteratively performed

(3) $$\left\{\begin{array}{c} h_{11}\left(t\right)=x(t)\\ h_{1k}\left(t\right)=h_{1\left(k-1\right)}(t)-m_{1k}(t),k>1 \end{array}\right)$$

k presented the number of detrending operation. *h*_1*k*_(*t*) was considered as an IMF only if the trend *m*_1*k*_(*t*)satisfies the criterion as the steady constant trend.

(4) $$h_{1k}\left(t\right)=\text{IM}F_{1}\left(t\right)$$

The first residue was computed.

(5) $$r_{1}\left(t\right)=x\left(t\right)-\text{IM}F_{1}\left(t\right)$$

The residue *r*_*i*_*(t)* was the target of ith iteration of EMD for *IMF*_*i*_*(t)* computation, and *x(t)* performed at the first iteration for *IMF*_1_(t). After n iteration, *x(t)* was decomposed into n IMFs, *IMF*_1_(t) ~ IMF_*n*_(t), and one residue, *r*_*n*_(*t*), which was either the steady trend or a constant.

(6) $$x\left(t\right)=\sum_{i=1}^{n}\text{IM}F_{i}\left(t\right)+r_{n}\left(t\right)$$

EMD decomposes the nonstationary data into finite set of IMFs without information loss or distortion. This study adopted mean-value criterion [8] with value 0.2 as stop criterion of IMF determination for the ABP signals decomposition. The extracted intrinsic characteristics were tested by orthogonality test and significant test [14]. It had been examined that the artifacts and unexpected high frequency influences were eliminated from ABP as one IMF, and the significant test was applied to distinguish the intrinsic noise characteristics [14].

### Instantaneous period based on normalized Hilbert transform

The instantaneous period is evaluated by the inverse of the instantaneous frequency. The instantaneous frequency presents how frequent the oscillation is at specific instant, and can be evaluated by angular velocity of the projection of source data *x(t)* on complex plane. NHT is a reliable method to project the source data onto complex plane. NHT contains two steps. First, the frequency modulation (FM) of source data was extracted by normalization process [15]. Then, the complex pair of FM part *x*_*FM*_*(t)* was calculated by Hilbert transform *H*(*x*_*FM*_(*t*)).

(7) $$y\left(t\right)=H\left(x_{\text{FM}}\left(t\right)\right)=\frac{1}{\pi }P.V.\int_{-\infty }^{\infty }\frac{x_{\text{FM}}\left(\tau \right)}{t-\tau }\mathrm{d\tau}$$

(8) $$e^{\mathrm{j\omega}\left(t\right)}=x_{\text{FM}}\left(t\right)+\text{jy}\left(t\right)$$

The instantaneous frequency was obtained by the differential of the angular velocity function *ω*(t), which was calculated from the complex composition of *x*_*FM*_*(t)* and its complex pair *y(t)*. The orthogonality was tested by orthogonality test and complex plane verification [8].

(9) $$\omega \left(t\right)=\tan^{-1}\frac{y\left(t\right)}{x_{\text{FM}}\left(t\right)}$$

(10) ω't=xFMty't−ytxFM'txFMt2+yt2

### Instantaneous pulse rate variability (iPRV)

**Figure 1:**
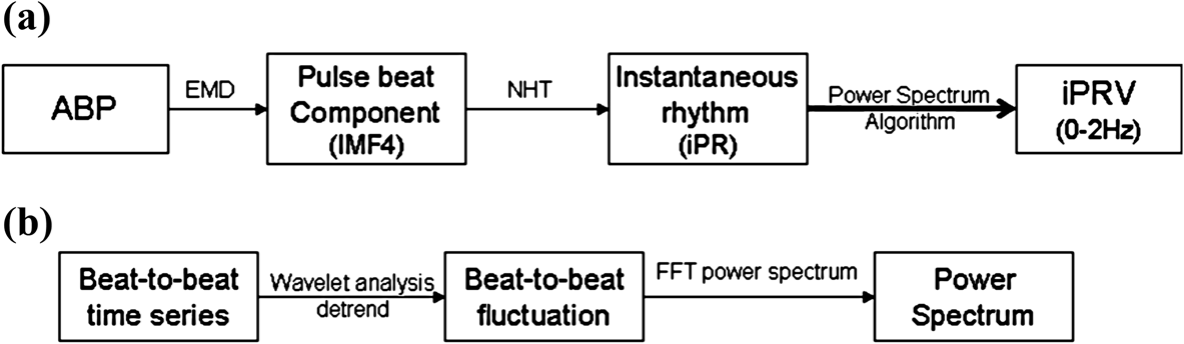
**The flow chart of analysis methods.** The methods include **(a)** instantaneous pulse rate variability (iPRV) and **(b)** power spectral analysis.

**Figure 2:**
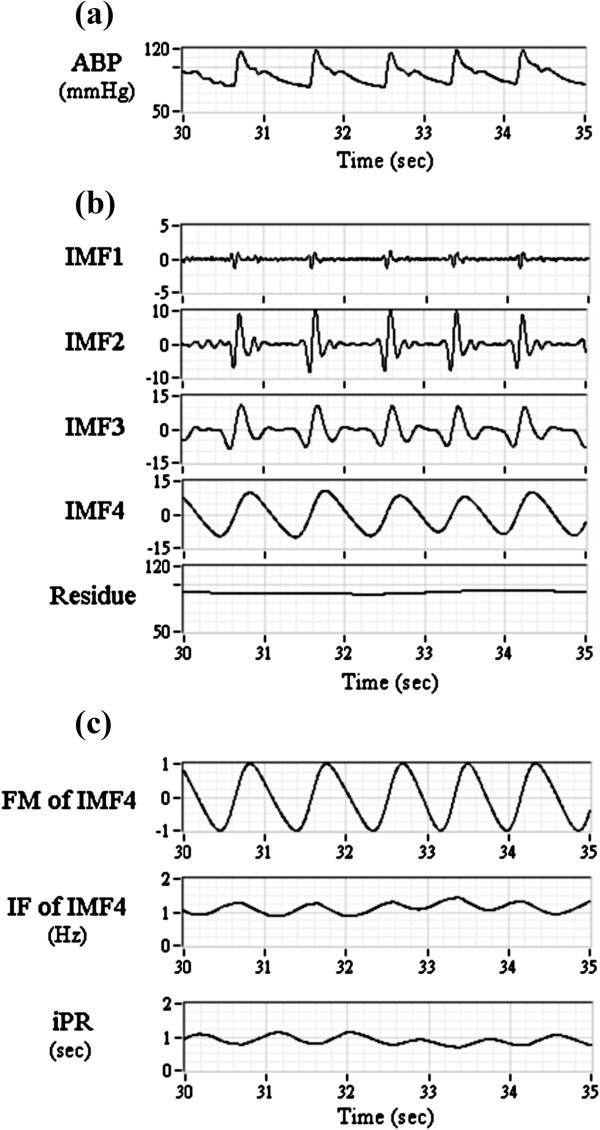
**Results of empirical mode decomposition (EMD) and normalized Hilbert transform (NHT) of arterial blood pressure (ABP) waveform.** It includes **(a)** original ABP waveform, **(b)** intrinsic component of ABP (IMF1-4 and residue), **(c)** frequency modulation (FM) of IMF4, instantaneous frequency (IF) of IMF4, and instantaneous pulse rate (iPR) within 5 seconds for example.

The steps of iPRV were illustrated in Figure 1. The pulse beat component (IMF4 in this study) was extracted from ABP waveform based on EMD [14], and its instantaneous oscillation, known as instantaneous period (iPR), was evaluated by NHT (Figure 2). The wavelet analysis was applied for the detrend process as constant trend elimination (<0.01 Hz). The amplitude modulation of pulse beat component was eliminated obviously after normalization process (Figure 2c). FFT spectrum was applied for power spectral estimation in each frequency band, including LF, HF, and VHF. The iPRV analysis program in this study was developed by using commercial software (LabVIEW v.2011, National Instruments Corp., Austin USA).

### Statistic analysis

Paired-sample t test was used to test the significance of the difference between baseline and the physiologic challenges, including 6-cycle breathing, 30-cycle breathing, and HUT, in each frequency bands in all subjects. P value of <0.05 was considered statistically significant. All statistical analysis was performed by using commercial statistics software (Statistical Package for Social Science, version 15.0, SPSS Inc., Chicago, Illinois).

## Results

**Figure 3:**
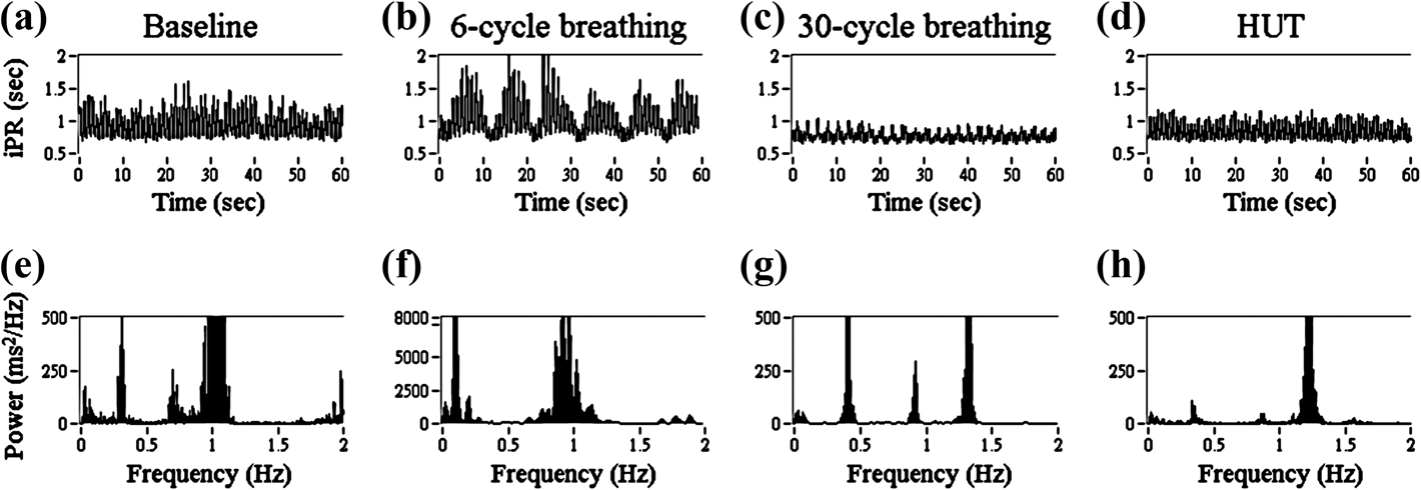
**The illustration of iPR series and corresponding fast Fourier transform (FFT) spectrum within 1 minute in one subject for example.** The physiologic challenges of iPR include **(a)** baseline, **(b)** breathing with 6 cycles per minute, **(c)** breathing with 30 cycles per minute, **(d)** head-up tilt (HUT) and corresponding FFT spectrum during **(e)** baseline, **(f)** breathing with 6 cycles per minute, **(g)** breathing with 30 cycles per minute, **(h)** HUT.

iPR series and corresponding FFT spectrum, called iPRV spectrum, are illustrated in Figure 3 with different physiologic challenges in one subject for example. The results of all subjects’ iPRV spectrum were similar with subtle change of the frequency peaks’ locations. The frequency range of iPRV spectrum was wide and contained several spectral characteristics above 0.4 Hz (Figure 3e), which exceeded the conventional frequency range of PRV. During 6-cycle breathing, the 0.1 Hz intrinsic oscillation of iPR was observed obviously, and the change of iPR amplitude was sharp compared with the other states (Figure 3b). There were two major spectral peaks in iPRV spectrum during 6-cycle breathing (Figure 3f). One was around 0.1 Hz and the other was around 1 Hz. The magnitude of these two spectral peaks was large. During 30-cycle breathing, iPR contained the lowest intrinsic trend, and the change of its amplitude was the smallest (Figure 3c) in all the states. There were four major spectral peaks in iPRV spectrum during 30-cycle breathing, including 0.1 Hz, 0.4 Hz, 0.9 Hz, and 1.3 Hz (Figure 3g). During HUT, the pulse amplitude of iPR series was smaller than it in baseline, and the locations of the major spectral peaks in iPRV spectrum were similar with those in baseline (Figure 3h). The power density during HUT was much lower than it in baseline.

**Figure 4:**
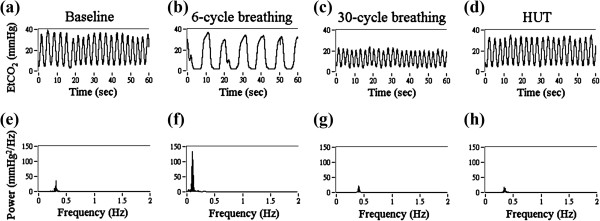
**The illustration of end-tidal CO**_**2 **_**(EtCO**_**2**_**) concentration and corresponding FFT spectrum within 1 minute in one subject for example.** The physiologic challenges of EtCO_2_ include **(a)** baseline, **(b)** breathing with 6 cycles per minute, **(c)** breathing with 30 cycles per minute, **(d)** head-up tilt (HUT) and corresponding FFT spectrum during **(e)** baseline, **(f)** breathing with 6 cycles per minute, **(g)** breathing with 30 cycles per minute, **(h)** HUT.

The respiration signal (EtCO_2_) and corresponding FFT spectrum are illustrated in Figure 4 with different physiologic challenges. The amplitude of EtCO_2_ was much larger during 6-cycle breathing (Figure 4b) and was much smaller during 30-cycle breathing (Figure 4c) compared to baseline (Figure 4a). The main frequency of the respiration was observed in FFT spectrum. The locations of the major spectral peaks were found at the same locations with those in iPRV spectrum. The statistical analysis of iPRV spectrum was summarized in Table 1. The power of LF increased statistically significant during 6-cycle breathing. The power of HF decreased statistically significant during HUT. The power of VHF increased during 6-cycle breathing and 30-cycle breathing, but decreased during HUT.

**Table 1:** Spectral analysis of instantaneous pulse rate variability (iPRV) during different physiologic challenges

	**LF (ms**^**2**^**)**	**HF (ms**^**2**^**)**	**VHF (ms**^**2**^**)**
Baseline	1161+/−1841	2223+/−2563	1258+/−1244
6-cycle	8522+/−5865§	1468+/−1424	2080+/−2395
30-cycle	897+/−822	898+/−1021	2333+/−2346^§^
HUT	802+/−624	1196+/−1397*	939+/−671

Present form is mean +/− SD with n = 15; *means p < 0.05 compared with baseline; ^§^means p < 0.01 compared with baseline.

LF denotes low frequency band (0.04-0.15 Hz); HF denotes high frequency band (0.15-0.4 Hz); VHF denotes very high frequency band (0.4-0.9 Hz); 6-cycle denotes paced respiration with 6 cycles per minute; 30-cycle denotes paced respiration with 30 cycles per minute; HUT denotes head-up tilt.

## Discussion

HHT contains the adaptive filter bank (EMD) and projects the physical oscillation onto complex plane with instantaneous frequency estimation (NHT) [8,15,16]. HHT provides a reliable way to evaluate the nonstationary intrinsic characteristics of source data, especially for biomedical signal. It had been examined that the pulse beat component can be extracted from ABP waveform based on EMD and corresponding instantaneous period, called iPR, can be estimated by using NHT. HHT improves the temporal resolution of PR series and helps to extend the frequency range of PRV spectrum, known as iPRV spectrum. iPRV spectrum has consistent result with PRV spectrum in conventional frequency bands, including LF and HF, and provides wide frequency range (0 ~ 2 Hz) for PRV spectral analysis [8].

iPRV spectrum contains four frequency bands of non-rhythmic neural regulatory in cardiovascular circulation in short-term ABP recording, including VLF, LF, HF, VHF. VLF is much less defined in short-term recording. LF presents both of SNS activities and PNS activities. Most of HF is coupling spontaneous respiration frequency (0.1-0.3 Hz) and presents the interaction between PNS, respiration, and cardiorespiratory coupling. VHF is a new frequency band of PRV, which may be related to high frequency fluctuations caused by reflex regulation and needs further investigation. The changes of power density in these frequency bands provide a quantitative measurement for autoregulation estimation. The major pulse rate was indicated by the spectral peaks above 1 Hz with the largest magnitude of spectral power in iPRV spectrum.

The respiration-related spectral peaks were observed at the consistent locations in iPRV spectrum with those in respiration spectrum. The magnitude of the respiration-related spectral peak was large during 6-cycle breathing (Figure 4f) and during 30-cycle breathing (Figure 4g). While the participants performed the paced respiration, the ABP was influenced by respiration in thorax, which was originated from the change in venous return owing to the change in intra-thoracic pressure, and it induced the reflex, including the modulation between SNS and PNS. The autoregulation induced by respiration would further influence the cardiac pumping function and peripheral resistance.

The power of LF (0.04-0.15 Hz) increased statistically significant during 6-cycle breathing and the main spectral peak was located around 0.1 Hz. It indicated the respiration-related intrinsic oscillation induced by 6-cycle breathing. The power of LF presents both of SNS activities and PNS activities. Both of these two ANS activities increased, which induced by 6-cycle breathing, and were indicated in LF. The respiration frequency was observed both in iPRV spectrum (Figure 3f) and in respiration spectrum (Figure 4f). The power of HF (0.15-0.4 Hz) increased statistically significant during 30-cycle breathing and the main spectral peak was located around 0.5 Hz. It indicated the respiration-related intrinsic oscillation induced by 30-cycle breathing. Moreover, the power of VHF increased both in two respiratory maneuvers. Though the differences of the VHF power between baseline and two respiratory maneuvers were not statistically significant with various individual differences, the results still suggested that a part of the autoregulation induced by respiration was indicated in VHF. The main frequency of dominant spectral peak in VHF is much complicated individually. The results emphasized that VHF has potential to indicate the autoregulation, which is neither the harmonic of respiration frequency nor the combination of pulse rate and respiration frequency. The paced respiration dominates one of the intrinsic oscillations of iPR at respiration frequency with complex regulatory mechanisms and the dominant frequency in VHF needs further examination.

The decreasing power of HF indicated the decreasing PNS activities with increasing SNS activities for the compensation of temporary postural hypotension during HUT. The power of VHF decreased during HUT and it suggested that VHF was influenced by the activities of ANS at higher frequency range, such as cardiac pumping function, the influence of cardiac performance related to respiration [9], and other cardiovascular autoregulation mechanism, such as baroreflex or peripheral vascular tone in nonstationary conditions. However, pulse rate was affected by peripheral regulation, such as arterial elasticity and peripheral resistance. These regulations might present by other intrinsic oscillations at specific frequency band in iPRV spectrum, which needs further investigation. The potential usefulness of VHF in the cardiovascular disease diagnosis also needs further examination. Literature examined that VHF of HRV provides a reliable index of cardiovascular disease [10], but the index is statistically significant while the heart rate is up to 120 bpm, which means that the index is only available during exercise. This study provided a reliable index (VHF of iPRV) for the evaluation during any nonstationary conditions and it is much convenient for the exploration of cardiovascular function. Besides, it had been examined that PRV can be measured by plethysmography or pulse oximetry, which is much convenient, simpler and cheaper than ABP measurement, and it makes iPRV have much potential usefulness of homecare monitor.

Though iPRV spectrum provides more information with wide frequency range, it has some limitations. First, the precise iPR is on the promise of that every blood pulse generated successfully by each cardiac muscle contraction, which might fail in some specific circumstances, such as premature ventricular contractions. Second, the precision of iPR depends on ABP measurement, such as pulse plethysmography. ABP measurements are sensitive to patient and probe-tissue movement artifacts and the sensing equipment is much elaborate. Though EMD, as the filter bank, has the capability to deal with high frequency noise caused by movement artifact, the automatic artifact detection and the replacement of corresponding corrupted signal segments are essential if the artifact breaks the morphology of the blood pulse waveform [17]. Third, iPRV depends on HHT. The parameters setting of HHT would mainly influence the results.

This study demonstrated a nonstationary approach to explore the intrinsic physical phenomena through intrawave frequency modulation. The method has potential to explore nonstationary phenomena through relevant biomedical signals, such as acoustic signal, electronic signal or respiration signal. But the indication is only reliable after serial verifications, including correctness of recording signal, parameters selection of HHT, orthogonality of IMFs extracted by EMD and after NHT, and significant test of the distribution. Besides, the frequency domain projection through NHT is only available while the target signal is sinusoidal-like signal [15], which means that the integrated electronic signal, such as electrocardiography (ECG) or electroencephalography (EEG), need specific normalization method and another projection function while applying this method.

## Conclusion

This study showed that iPRV spectrum provided the wide frequency band for non-rhythmic regulation estimation. The frequency bands in iPRV spectrum include VLF, LF, HF, VHF, and higher frequency band coupling pulse rate. The certain frequency band changes during different respiratory maneuvers and HUT. The new frequency band, known as VHF (0.4-0.9 Hz), was influenced by respiration and HUT. The dominant frequency in VHF is neither the respiration frequency nor the combination of pulse rate and respiration frequency. During different respiratory maneuvers, the power of VHF increased, which was mainly due to the influences of cardiorespiratory coupling. During HUT, the power of VHF decreased, which was mainly due to the influences of the modulation between SNS and PNS. iPRV provides a nonstationary approach to explore ANS function. Moreover, the new frequency band (VHF) provides the potential indication of cardiorespiratory coupling and autoregulation estimation. It helps for intrinsic characteristics exploration and quantitative evaluation of cardiovascular autoregulation.

## Abbreviations

ANS: Autonomic nervous system; HRV: Heart rate variability; PR: Pulse rate; PRV: Pulse rate variability; iPRV: Instantaneous pulse rate variability; HUT: Head-up Tilt; FFT: Fast Fourier transform; DWT: Discrete wavelet transform; LF: Low frequency; HF: High frequency; SNS: Sympathetic nervous system; PNS: Parasympathetic nervous system; iPR: Instantaneous pulse rate; HHT: Hilbert-Huang transform; VHF: Very high frequency; ABP: Arterial blood pressure; EtCO2: End-tidal CO_2_; NHT: Normalized Hilbert transform; FM: Frequency modulation; IMF: Intrinsic mode Function; EMD: Empirical mode decomposition; ECG: Electrocardiography; EEG: Electroencephalography.

## Competing interests

The authors declare that they have no competing interests.

## Authors’ contributions

CC: made the substantial contributions to the conception, design, analysis and interpretation of data, and has drafted the manuscript. HY: made contributions to the experiment design, perform the clinical trials approved by institutional review board, and interpretation of data. TC: the corresponding author, made contributions to the conception and analysis method, have given the final approval of the version to be published. All authors read and approved the final manuscript.

## Authors’ information

CC, the first author, received the M.S. degree in Biomedical Engineering and Ph. D. degree in Computer Science and Engineering from the National Chiao Tung University, in 2010 and in 2013, respectively. Currently, he is assistant research fellow at Biomedical Electronics Translational Research Center (BETRC) and Biomimetic Systems Research Center (BSRC) in National Chiao Tung University, Hsinchu, Taiwan. His research interests include bioinformatics, biomedical signal processing, and pattern recognition. HY received the M.D. degree from the School of Medicine, National Yang Ming University, Taipei, Taiwan, in 1991, and the Ph.D. degree in Clinical Medicine from the Institute of Clinical Medicine, National Yang Ming University in 2004. He is currently an Associate Professor with the School of Medicine, Chung Shan Medical University, Taichung, Taiwan. He is an attending physician in Neurology, Tungs’ Taichung MetroHarbor Hospital, Taichung, Taiwan. His major research interests include cerebrovascular diseases, diagnostic ultrasound, cerebrovascular regulation, and biomedical signal processing. TC, the corresponding author, received the M.S. degree in Physics and Ph.D. degree in Biomedical Engineering from National Sun Yat-Sen University and National Yang-Ming University, Taiwan, in 1996 and in 2003, respectively. In 2003, he joined the faculty of the Department of Biomedical Engineering at I-Shou University, and now he is an assistant professor of Department of Computer Science, Institute of Biomedical Engineering, National Chiao Tung University (since 2006), and Biomedical Electronics Translational Research Center (BETRC) and Biomimetic Systems Research Center (BSRC). His research interests include neural networks, virtual biomedical instrumentation (VBI), and multivariate spectral analysis.
